# Infection, vaccination and risk of dementia: a proposed immunological model

**DOI:** 10.3389/fimmu.2026.1748535

**Published:** 2026-03-04

**Authors:** Justin Devine, Bart Jacobs, Isabel Leroux-Roels, Geert Leroux-Roels, Robbert van der Most

**Affiliations:** 1 Synexa Life Sciences, Cape Town, South Africa; 2 Center for Vaccinology (CEVAC), Ghent University and Ghent University Hospital, Ghent, Belgium; 3 VaxxCellence, Antwerp, Belgium

**Keywords:** AS01 adjuvant, BCG - Bacille Calmette-Guérin vaccine, dementia, neuro-inflammation, shingles (herpes zoster) vaccine, trained immunity

## Abstract

With ageing populations, the prevalence of different types of dementias is increasing. The pathology of Alzheimer’s disease (AD), the most common form of dementia, has been linked to the presence of plaques and neurofibrillary tangles in the central nervous system of patients. There are growing indications that risk of developing dementia correlates with several infectious agents, including human herpes viruses, flaviviruses and SARS-CoV-2. This has led to a proposition that AD and other dementias could be considered as having an infectious disease etiology. Whilst the mechanisms behind this remain unclear, intriguing epidemiological data suggest that several vaccinations are correlated with *reduced* risk for dementia. Intravesicular administration of the tuberculosis vaccine strain Bacille Calmette-Guérin (BCG) has been associated with decreased risk of dementia in bladder cancer patients. This has led to the hypothesis that non-specific effects of vaccinations, mediated through trained innate immunity, provide a mechanistic explanation. Over the last few years, the AS01-adjuvanted recombinant shingles vaccine has also been associated with reduced risk in several studies. Moreover, in a recent study, immunization with the adjuvanted RSV vaccine, also containing AS01, was shown to reduce risk of dementia. Integrating data on BCG and mechanistic hypotheses, recent findings on the AS01 adjuvant, and the role of trained innate immunity, we describe here an immunological model that connects vaccine and adjuvant mode of action with risk of dementia. This immunological model can help shape a research roadmap to further elucidate the mechanisms behind the collective epidemiological data.

## Introduction

According to WHO data, 57 million (mio) people worldwide suffered from dementia in 2019 (1), with Alzheimer’s disease (AD), the most common form of dementia, accounting for 60-70% of these cases (1). About 60% of individuals with dementia live in low- and middle-income countries, and there are approximately 10 mio new cases each year. European case numbers were 9.78 mio across countries represented by Alzheimer Europe members, and are expected to almost double to 14.3 mio (EU27) or 18.8 mio (wider Europe) cases by 2050 (2). With the global population aging rapidly, there is an urgent need to develop new treatment options or modalities aimed at preventing neuro-degenerative disease.

Of note, most of the studies evaluating the effects of infection and vaccination on dementia, as discussed below, have focused on an all-cause etiology and diagnosis of clinical dementia, which includes AD and other types of dementia such as vascular dementia and Lewy Body dementia, with definitions varying across studies. For this reason, the term ‘dementia’ will be used here to cover all the different etiologies.

The exact pathophysiological mechanisms underlying AD or other forms of dementia, such as vascular dementia and Lewy Body dementia, are not fully elucidated. However, increasing evidence suggests that infections may contribute to the development of dementia, including amongst others AD. Viral pathogens related to such dementia-associated infections include neurotropic viruses, such as human herpesviruses, as well as flaviviruses and SARS-CoV-2 (3–5). Consistent with this, Duggan et al. identified various infections, including those with human herpesviruses, but also upper and lower respiratory tract infections, as being associated with different levels of neurodegeneration risk (6). For these reasons, a viral infection etiology of dementia, as first proposed by Oskar Fischer in 1907, is receiving more attention (4, 7, 8). However, the potential roles of bacterial infections and gut microbiome dysbiosis have also been highlighted (6). A recent review identified both viral and bacterial infections as dementia-risk increasing, including, but not limited to, human herpesviruses, periodontal disease, viral encephalitis and bacterial meningitis (9). A systematic analysis of the associations between the prescribed drugs and patients’ dementia risk identified antibiotics and anti-inflammatory drugs with a reduced risk, and diabetes drugs and antipsychotics with an increased risk (10).

The hypothesis that human herpesviruses such as Varicella Zoster Virus (VZV) and Herpes Simplex Virus (HSV) may be involved in the initiation and/or progression of dementia—in part supported by the effects of anti-herpesvirus drugs (9, 11)—has received considerable attention (4, 5, 8, 12–14). In fact, one hypothesis is that prevention of VZV reactivation could be mechanistically linked to reduced risk of dementia (13). Indeed, treatment with anti-herpesvirus drugs has been associated with decreased risk of dementia (15). However, a recent study assessing the impact of the anti-herpesvirus drug valacyclovir on dementia progression in older adults diagnosed with early dementia did not demonstrate a significant effect (16), suggesting that while there may be a preventive effect, antiviral drugs are not effective as therapeutic agents against dementia.

Interestingly, a systematic review and meta-analysis by Han et al. (17) on the association between intravesicular Bacille Calmette-Guérin (BCG) vaccine administration and risk of dementia in bladder cancer patients postulated that BCG treatment resulted in 45% decreased risk of dementia in these patients (17). Although selection bias in the included studies could not be ruled out due to their retrospective nature, sensitivity analysis confirmed the robustness of the data (17, 18). This has led to the hypothesis that non-specific effects of vaccination, operating through trained innate immunity, could provide a mechanistic explanation of the observed reduced risk of dementia (19). Interestingly, novel data suggest that the trained immunity mechanisms demonstrated for BCG (20, 21) can be extended to two vaccines containing clinically relevant adjuvants (22, 23). Based on this data, we describe how the dementia-protective mechanisms proposed for BCG could be applied to other vaccines as well (18, 19, 21, 24).

Despite the increasing interest in an infectious disease etiology of dementia (4, 6, 8), it should be emphasized that this etiology is multifactorial and complex, with important contributions from metabolic inflammation, the gut-brain axis and vascular pathology. The intricate associations between systemic inflammation, the microbiome, neuroinflammation and dementia were recently reviewed by Xie et al. (25). The interconnected roles of metabolic inflammation and cognitive decline are evidenced by metabolic syndrome serving as a significant risk for, primarily, vascular dementia (26–30). Furthermore, dementia patients often exhibit gut microbiome dysbiosis with reduced microbial diversity, a decrease in beneficial bacteria such as *Eubacterium rectale, Bifidobacterium*, and *Dialister*, and an increase in pathogenic bacteria including *Escherichia/Shigella, Bacteroides*, and *Ruminococcus* (31). Dysbiosis of the gut microbiome appears to be a key driver of neuroinflammation, acting by disrupting the gut barrier integrity, promoting systemic inflammation, and intensifying neuroinflammatory responses, thereby accelerating dementia progression (25, 32). Gut microbiota-derived metabolites (e.g., short-chain fatty acids and lipopolysaccharides) directly influence microglial activation and amyloid aggregation (33). Indeed, the connections between lipid metabolism and neuroinflammation (34), including the metabolic and epigenetic reprogramming of myeloid cells, link the roles of diet, the apolipoprotein E (*APOE*) ϵ4 allele, the microbiome, and innate training, with neuroinflammation. Aligned with these data, diet-induced dysbiosis results in long-term NLRP3 inflammasome-mediated epigenetic reprogramming of innate immune cells, in turn resulting in chronic systemic inflammation (35). Microglial activation of NLRP3 by β-amyloid and Tau in dementia increases the level of reactive oxygen species in the brain, which in turn aggravates mitochondrial dysfunction and impairment of autophagy (36). This could accelerate both neuroinflammation and the resulting neuronal damage in the central nervous system (CNS) (36).

## Vaccination can reduce risk of dementia

Beyond BCG, several routine vaccinations including those with the combined tetanus/diphtheria/acellular pertussis (Tdap), tetanus/diphtheria (Td), hepatitis A, pneumococcal and influenza vaccines, have been associated with a decreased risk of dementia (10, 37, 38). A meta-analysis of the association between influenza vaccination and risk of dementia, covering eight studies and 9.9 mio individuals, confirmed a dose-dependent dementia risk reduction in high-risk populations (39). However, opposing effects of vaccination have also been reported: a cohort study of 13 mio individuals ≥50 years of age revealed an increased risk of dementia associated with vaccination (with an adjusted odds ratio of 1.38) (40), illustrating the complexity of this issue as well as the caution needed to interpret the epidemiological data. This effect appeared to be primarily driven by influenza and pneumococcal vaccines, with no significant effects reported for the adjuvanted shingles, tetanus, diphtheria and pertussis vaccines (40). However, as reported by the authors and in a subsequent commentary (41), unmeasured confounding and a late-detection bias could have played a role in the observed risk increases. It is noteworthy that BCG was not included in this study (18) and that only <6% of the study population had received a vaccination with the adjuvanted shingles vaccine (41). As a result of the collective data, interest in the associations between vaccination and dementia risk is increasing (24).

Recently, epidemiological evidence has emerged that vaccination with shingles vaccines confers protection against dementia in older adults (37, 38, 42–45). The live-attenuated zoster vaccine (ZV), used to prevent shingles, was shown to be associated with a reduced dementia risk in adults ≥70 years of age, with an adjusted hazard ratio (HR) of 0.72 during a 7-year observation period (44). Interestingly, the authors concluded that there was no evidence that the association between shingles vaccination and dementia “*was mediated by a reduction in shingles diagnosis”*. This conclusion was informed by the fact that the ZV-vaccinated individuals with or without shingles diagnosis had very similar HRs as compared to unvaccinated individuals without shingles, i.e. 0.69 and 0.71, respectively (44). A large-scale claims-based database study revealed a 32% risk reduction associated with vaccination with the AS01-adjuvanted recombinant zoster vaccine (RZV) (46). In another study of adults aged ≥65 years, receipt of at least one dose of the live-attenuated shingles vaccine was associated with a 7.3% risk reduction over an 8-year period, whereas receipt at least one dose of the RZV vaccine was associated with a 72% risk reduction over a 2-year period (37). These differences may reflect variations in shingles vaccine efficacy; alternatively, the AS01 adjuvant in the RZV vaccine may directly modulate dementia risk. In contrast, a recently published longitudinal analysis of health records from >100 mio individuals in the US, revealed a lower risk of dementia associated with vaccination with either shingles vaccine as compared to 23-valent pneumococcal polysaccharide vaccine, with dementia risk reductions of 33% and 27% for the live-attenuated vaccine and RZV, respectively, over a 3-year period (13). A systematic review and meta-analysis on the association between herpes zoster (HZ) infection, antivirals and vaccination and the risk of developing dementia, covering 18 studies and 9.4 mio individuals, confirmed an increased risk associated with HZ infection (Relative Risk [RR] = 1.14) and protective effects of both antivirals (RR = 0.84) and vaccination (RR = 0.68) (14). In a very recent study, Xie et al. demonstrated, using data from Wales and Australia and a quasi-randomized design, that vaccination with the live-attenuated shingles vaccine “*also reduces mild cognitive impairment diagnoses and, among patients living with dementia, deaths due to dementia*” (47); amongst the limitations, under-ascertainment of mild cognitive decline has been highlighted (47). Also, the study did not include the adjuvanted RZV vaccine as this vaccine was introduced after the observation period.

Taquet et al. (48) reported that *both* RZV/AS01 and the AS01-adjuvanted prefusion F (preF)-based RSV vaccine, named RSVPreF3/AS01, administered individually or combined, were associated with reduction of the risk of dementia diagnosis over an 18-month observation period in older adults (48). Compared to seasonal influenza virus vaccination, the RSV and RZV vaccines reduced the 18-month dementia risk by 29% and 18%, respectively, with a 37% reduction observed when both vaccines were combined. No differences in risk reduction were noted between the two AS01-adjuvanted vaccines in this study (48). This led the authors to conclude that “*the AS01 adjuvant itself plays a direct role in lowering dementia risk*” *(*48). These findings regarding RZV (48) are consistent with previous data and with the summary data from two industry-sponsored epidemiological studies examining the association between RZV vaccination and dementia risk in older adults in the US (49). However, as acknowledged by the authors (48, 50, 51), interpretation of the results was complicated by the lack of a clear adjuvant dose-response effect in the dataset, as the RSVPreF3/AS01 vaccine contains half the adjuvant dose as compared to RZV/AS01 and is given in one dose as compared to two doses for RZV/AS01. In a recent commentary (51), it was argued that the Taquet et al. study (48) failed to provide evidence for a direct role for the adjuvant, because 44% of the RSV vaccinations in the study were not brand specified, thus including both the bivalent nonadjuvanted preF-based RSV vaccine and the RSVPreF3/AS01 vaccine. Therefore, these authors reasoned that a role of prevention of RSV infection could not be excluded (51), which led to a response arguing that both adjuvant-driven and pathogen-prevention mechanisms could play a role (50).

It should be noted that the collective epidemiological data referred to here have the strengths and limitations inherent in observational studies. Prominent amongst these are selection bias and differences in healthcare seeking behavior between individuals that were vaccinated and individuals that were not. However, the natural experiment as conducted by Taquet et al. reduces unmeasured confounding (50). The study by Eyting et al. mitigates these typical observational biases by utilizing a regression discontinuity design—a unique natural experiment based on an exact date-of-birth eligibility threshold—which provides evidence that is less vulnerable to confounding and selection bias than existing associational studies. By comparing individuals born just days apart, the researchers created a ‘quasi-randomization’ where both observed and unobserved factors, such as healthcare-seeking behavior and personal motivation, were balanced across the groups (43).

While diverging data has been published for the different vaccines, as discussed above, the overall trend for the VZV vaccines appears to be supportive of a protective effect.

## An immunological model connecting the opposite effects of vaccination and infection on dementia development

The epidemiological observations described above raise the need to elucidate the potential mechanism(s) by which the risk of dementia either increases or decreases in association with infection or its prevention respectively. The reduced risk observed with intravesicular BCG administration (18, 19, 21) has been mechanistically linked with trained innate immunity, based on the pioneering work by Netea and coworkers (20, 52). The observations that both the AS01-adjuvanted RSV and RZV vaccines, as well as Tdap and other vaccines have shown protective effects, are aligned with a potential role of non-specific effects of vaccination described for BCG (18, 19, 21). Furthermore, if an infectious etiology or risk factor underlies the dementia risk, it is likely to be multi-factorial. The protective effect of anti-inflammatory drugs (10) is consistent with the view that neuro-inflammation is a key immunological parameter upon which the opposing risk profiles associated with different infectious agents, vaccines, and anti-inflammatory or antiviral drugs, mechanistically converge (19, 25). From this perspective, a recently described transcriptomic signature of immune dysregulation— associated with infection severity, chronic diseases and all-cause mortality, and modifiable through immune-modulatory drugs—may be relevant as a potential biomarker (53). Finally, roles for both β-amyloid and Tau phosphorylation in innate immunity have recently been described (54–56). In summary, there are several connections between inflammation and risk of dementia. Chronic inflammation is known to disrupt cellular metabolism; consistent with this, restoring nicotinamide adenine dinucleotide (NAD+) homeostasis in a mouse model was shown to reverse *“tau phosphorylation, blood-brain barrier deterioration, oxidative stress, DNA damage, and neuroinflammation and enhances hippocampal neurogenesis and synaptic plasticity, resulting in full cognitive recovery and reduction of plasma levels of the clinical AD biomarker p-tau217*” (57).

**Figure 1:**
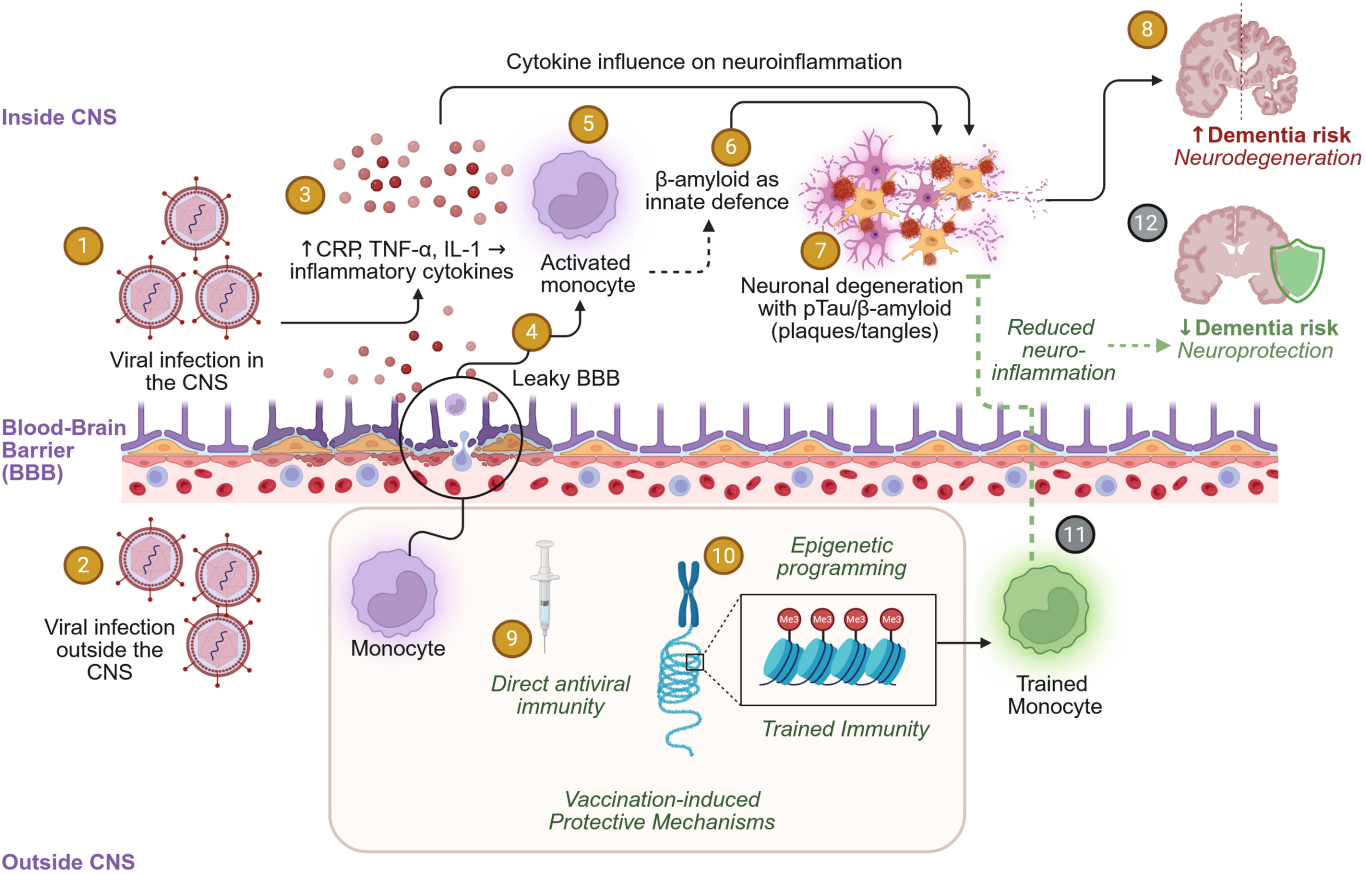
A hypothetical model connecting infection, vaccination and dementia risk. Viral infection either within (no. 1) or outside (no. 2) the central nervous system (CNS) could lead to innate immune activation. In turn, the innate immune responses result in upregulated production of pro-inflammatory markers such as C-reactive protein (CRP), TNF-α, and IL-1 (no. 3) in the CNS, and/or in changes in blood-brain barrier permeability (no. 4). The latter change can then result in increased susceptibility to other infections in the CNS as well as in activated peripheral innate immune cells entering the CNS (no. 5). Both β-Amyloid (6) and phosphorylated Tau (pTau) present in the CNS could play a role in innate immune responses (54–56) but also in pathogenicity (dementia development) (no. 7), potentially leading to neurodegeneration (no. 8). Conversely, certain vaccinations have direct antiviral effects (no. 9), and can also induce trained innate immunity through epigenetic reprogramming of innate immune cells (18, 21) (no.10). This epigenetic reprogramming operates through histone methylation at specific gene loci (23) (no.10). The resulting trained immunity including trained monocytes (no. 11) could then modify the local innate environment in the CNS. However, trained immunity could also result in non-specific antiviral effects of vaccination. Eventually, both the direct and the non-specific antiviral effects could play a role in reducing infection and inflammation burden in the CNS, thereby lowering the dementia risk (no. 12). Black arrows indicate risk increasing effects through increased neuronal inflammation. Green dashed arrows/inhibitor lines indicate the hypothetical vaccine-induced effects countering neuroinflammation and reducing risk. Steps 1–10 are supported by data, whereas steps 11 and 12 (indicated by grey numbers) are hypothetical. Created with biorender.com.

Based on the data and literature quoted above, we describe a hypothetical immunological model (**Figure 1**) in which (i) chronic or repeated viral infection (8), including herpesviruses (4, 5, 12) and respiratory tract infections (6), lead to elevated levels of (neuro)inflammation and systemic or dysregulated inflammation; (ii) vaccination reduces the total infectious disease burden; and (iii) non-specific effects of vaccination play a role counteracting chronic inflammation, extending the hypotheses brought forward to explain the BCG observations (18, 19, 21). As noted by several authors, there are strong data-sets demonstrating that neuroinflammation is associated with onset or progression of neurodegenerative disease (25, 58). This inflammation could trigger innate β-amyloid responses (54), consistent with the ‘Antimicrobial Protection Hypothesis’ where amyloid acts as an antimicrobial peptide (59), and Tau phosphorylation (56), given the roles of these proteins in innate immunity. This explanation is in fact consistent with the recently proposed hypothesis that the RZV vaccine reduces neuroinflammatory risk by suppressing silent episodes of VZV reactivation, thereby lowering the cumulative inflammatory exposure encountered by microglia in the CNS (58). Neuroinflammation also increases the permeability of the blood-brain barrier (25, 60) which could, in turn, lead to an increased burden of infectious disease and inflammation in the brain. Therefore, we hypothesize that vaccination may be associated with reduced risk because it decreases the cumulative burden of various infections and, consequently, the overall levels of neuroinflammation (**Figure 1**). Differences between vaccines could be explained by this multifactorial etiology—if different infections contribute to the risk, vaccination against any single infectious agent would only address part of the pro-inflammatory burden.

The additional explanation is based on a role for the non-specific effects of vaccination. Such non-specific effects, mechanistically linked to trained innate immunity (52), could affect the level of inflammation, and thus be involved in the overall protective effects of vaccination (**Figure 1**). As described for BCG, trained innate immunity is linked to epigenetic changes in innate immune cells, such as monocytes and their bone marrow progenitors, through chromatin modification of these cells (20, 52). These epigenetic changes result in innate immune memory, enabling modified or accelerated immune responses upon encountering new pathogens. It is now increasingly clear that these effects, which manifest in various forms, are not limited to BCG but extend to other vaccines and adjuvants as well (22, 23). For example, vaccination with seasonal influenza vaccines in healthy young adults induced epigenetic changes in monocytes and dendritic cells, leading to reduced inflammatory capacity, as evidenced by decreased expression of pro-inflammatory cytokines such as TNF-α, IL-1β, IL-12 and others (22). The addition of the oil-in-water emulsion adjuvant AS03 to an avian H5N1 influenza vaccine elicited similar effects as well as increased chromatin accessibility of interferon response factor (IRF) loci, possibly enhancing non-specific antiviral protection, as demonstrated in *in vitro* models (22). Similarly, the adjuvanted RZV and RSV vaccines (48) contain AS01 (consisting of the Toll-like receptor 4 ligand monophosphoryl lipid A [MPL] and QS-21 in a liposome formulation), and this adjuvant was shown to induce epigenetic modifications in monocytes (23). Indeed, it was demonstrated that administration of an AS01-adjuvanted hepatitis B surface antigen (HBsAg) vaccine to young healthy adults resulted in reduced accessibility of Activator Protein 1 (AP-1)-targeted loci, predicting an anti-inflammatory effect (23). AS01 also was associated with sustained (up to 6 months) increases in IRF locus accessibility, indicating potential non-specific antiviral protection. In this context, it is noteworthy that non-specific effects of vaccination with AS01-adjuvanted RZV against a heterologous infectious disease have been previously reported (61). A large-scale epidemiological cohort study showed that RZV vaccination conferred a 16% reduction of the risk of contracting COVID-19 and a 30% reduction in COVID-19 associated hospitalization of adults aged 50 years and older (61), suggesting a non-specific mechanism that protects against SARS-CoV-2 viral infection and disease progression. The authors argued (62) that the dual cohort design and the case-control test-negative design addressed comments on confounding and bias due to healthcare-seeking behavior (63). The effect was observed for less than 1 year, which aligns with the observation in young adults that the chromatin changes induced by the AS01-adjuvanted HBsAg vaccine remain detectable up to 180 days post-vaccination (23). Given the short lifespan of monocytes [approximately 1.0 ± 0.26 days (64)], the persistence of these chromatin modifications indicates that the progenitors in the bone marrow must also be subject to similar alterations (20, 52).

These trained immunity data suggest that RZV may exert its protective effects on dementia risk not only by preventing VZV reactivation and shingles, but also non-specifically, by protecting against other infections, as demonstrated by the COVID-19 data (61). It is important to note that current evidence on the epigenetic effects of these vaccine adjuvants is limited, and the impacts of age, the vaccine antigen itself, pathogen pre-exposure, the role of adaptive memory and other host factors remain unclear and warrant further research.

## Direct or indirect trained innate immunity?

The trained immunity mechanism offers an appealing theoretical framework for explaining how different vaccines might reduce dementia risk, particularly given that its etiology is unlikely to be attributable to a single pathogen. Trained innate immunity responses, as described for BCG (52), AS03 (22) and AS01 (23), may confer broader protection by reducing the overall infectious burden; we hypothesize that, as a consequence, this reduction could lower levels of neuro-inflammation (58). This mechanism could operate at two levels. First, it may involve reprogramming critical innate immune cells, such as monocytes and dendritic cells, toward a state of reduced or modulated inflammatory responsiveness. By diminishing neuroinflammation and enhancing interferon (IFN) responses, this process could confer increased antiviral protection against the pathogen in question (22, 23). Vaccination with adjuvanted vaccines may then create an innate environment that is more responsive to diverse pathogens by enhancing innate effector functions. Second, reprogrammed innate immune cells can function as antigen-presenting cells in a *de novo* response to a novel, unrelated pathogen, thereby accelerating the adaptive immune response to this pathogen and providing enhanced protection. These mechanisms are not mutually exclusive. However, further research is essential to translate epidemiological findings linking vaccination to reduced dementia risk into novel therapeutic or prophylactic strategies for dementia.

## A role for adaptive immune responses

**Figure 2:**
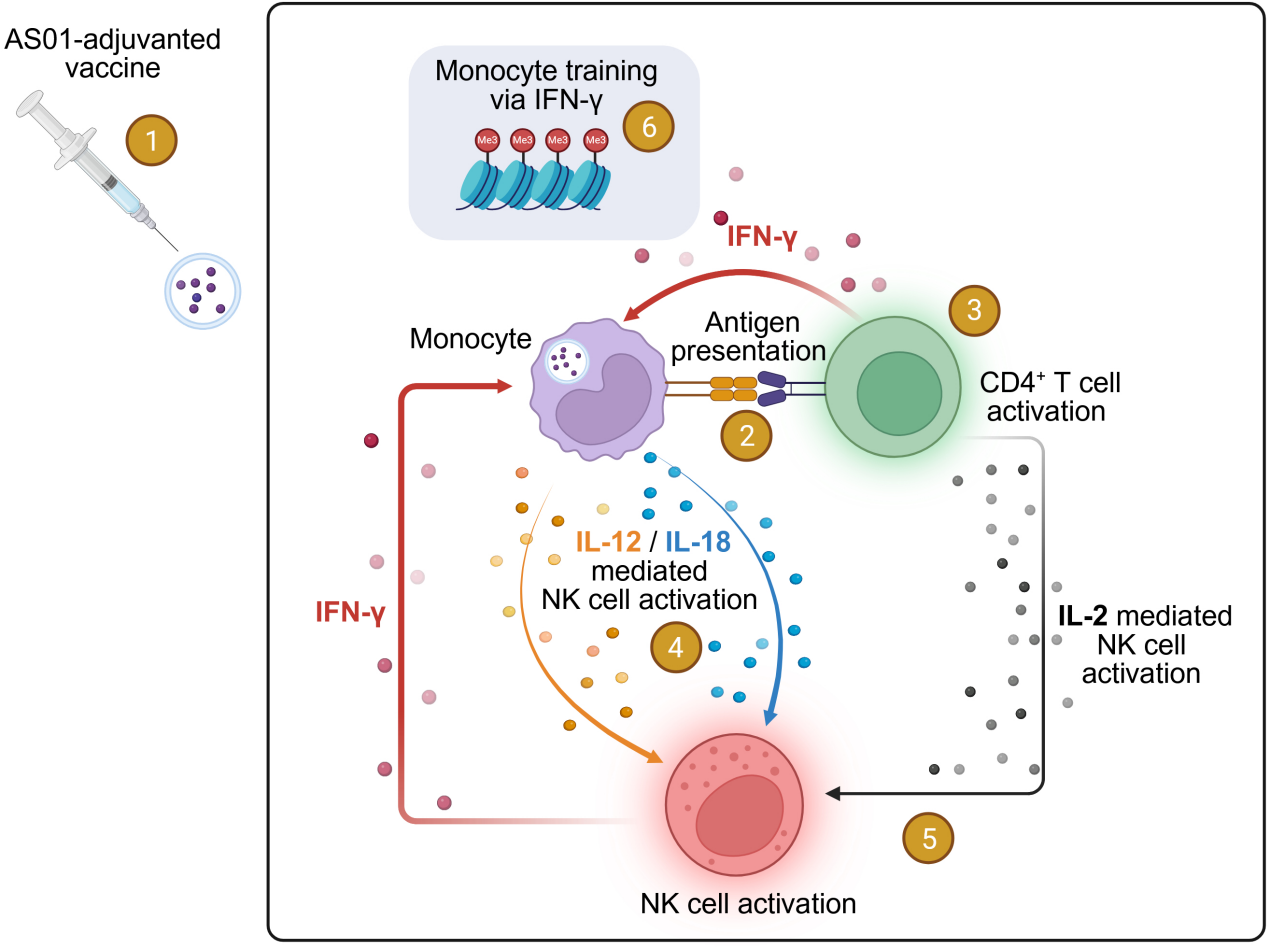
Innate/adaptive interaction and trained immunity upon vaccination. Mode of action data from AS01-adjuvanted vaccines (67, 69) and the *integrated organ immunity* hypothesis (65) point towards adaptive-innate feedback and a role for IFN-γ in monocyte/DC modification. Vaccination with an AS01-adjuvanted vaccine (no. 1) results in antigen presentation by innate immune cells such as dendritic cells and monocytes (70) (no. 2), which then activates CD4^+^ T cells (no. 3). Antigen-presenting cells also produce IL-12 and IL-18 cytokines, which activate NK cells (71) (no. 4). NK cell activation is amplified by IL-2 from CD4^+^ T cells (72) (no. 5). Both activated CD4^+^ T cells (no. 3) and activated NK cells (no. 5) can produce IFN-γ. The IFN-γ signal from NK and CD4^+^ T cells is hypothesized to further activate monocytes, and to provide a signal to stimulate the epigenetic modifications in these cells (66) (no. 6) that underlie trained innate immunity (65). Steps 1–5 are supported by data whereas step 6 is hypothetical and partially supported by data. Created with biorender.com.

Recently, the concept of ‘integrated organ immunity’ was proposed by Pulendran and coworkers (65, 66), for BCG and lung immune responses, which led the authors to suggest the possibility of a broadly specific or universal vaccine against multiple pathogens (65). We hypothesize that this concept could explain how different vaccines, in the current context particularly AS01-adjuvanted vaccines and BCG, may exert non-specific effects. Specifically, the hypothesis of integrated organ immunity posits that antigen-specific CD4^+^ T cells provide feedback signals to innate immune cells of the myeloid lineage, such as monocytes, potentially via IFN-γ, thereby delivering an epigenetic training signal (65). This process may be critical for regulating tissue-specific trained immunity. This model is consistent with previous data on innate immune responses induced by AS01- and AS03-adjuvanted HBsAg vaccines: innate responses following a second vaccine dose—when cognate memory CD4^+^ T cells are present—were significantly stronger than those observed in naive individuals after the first dose (67, 68) (**Figure 2**). In the context of the reduced dementia risk observed with AS01-adjuvanted RZV and RSV vaccines, it is conceivable that pre-existing cognate memory CD4^+^ T cells activated by vaccination, mediated the myeloid cell training signal, resulting in non-specific protection. This mechanism could, for example, explain the observed protection against COVID-19 (61) and may also extend to a reduced risk of dementia (42, 43, 48). Notably, this model of adaptive feedback on trained innate immunity may also account for the lack of an adjuvant dose-response effect reported in the recent study by Taquet et al. (48, 50, 51). If trained immunity depends in part on cognate CD4^+^ memory T cells, it can be hypothesized that the quality and quantity of CD4^+^ T cells specific for the vaccine antigens (i.e., RSVpreF3 and VZV gE) contribute to the overall effect.

## Discussion and summary of the hypothesis

The epidemiological data indicate that there is a connection between infections and vaccinations on the one hand and dementia on the other. The data show that infections increase risk of dementia (6, 8, 73), whereas vaccinations (14, 37, 39), particularly with BCG (17) and AS01-adjuvanted vaccines (48), decrease this risk, without clear pathogen-specific patterns. There are some conflicting data, particularly for influenza and pneumococcal vaccines, which may be attributable to late detection bias and unmeasured confounding (40, 41). Moreover, the attribution of the protective effects to the AS01 adjuvant, as has been proposed by Taquet et al, based on the RZV and RSV vaccine data, has been challenged (48, 50, 51). Nevertheless, two recent meta analyses of these data support the protective effects of influenza and HZ vaccination (14, 39).

Here, we propose that the concept of trained innate immunity, as developed to explain non-specific effects of BCG vaccination (17–19, 21, 52), provides a framework to explain this epidemiological data. A central element in this immunological model is that uncontrolled or excessive levels of neuro-inflammation, associated with elevated dementia risk, can be counteracted by epigenetic reprogramming of innate immune cells.

Thus, connecting the diverse epidemiological data (42, 43, 48, 61) with emerging adjuvant epigenetic data (22, 23), the microbiome/metabolic impact on neuroinflammation (25, 33), and the risk of dementia (**Figure 2**), leads to the following working hypothesis: certain vaccines, such as RZV and RSVPreF3/AS01, can induce both specific and non-specific protection, driven by the innate immune responses induced by adjuvants – which is close to the proposed mode of action of BCG (18, 21). This results in reductions of both the overall infectious disease burden and neuro-inflammation, mediated by myeloid cell reprogramming. The consequence of this would be a reduced involvement of β-amyloid- or phosphorylated Tau (pTau)-mediated innate immune responses in the brain (54, 55), translating into a reduced risk of dementia and delay in onset of symptoms.

The synthesis presented here has several limitations. First, accurate interpretation of the available data is constrained by a discrepancy between the timescales of non-specific effects mediated by trained immunity (typically, several months) and of the development of dementia (typically years to decades). Second, although the epigenetic changes associated with trained innate immunity occur at the level of myeloid progenitor (20), the link between systemic monocyte training and modulating inflammation within the CNS remains unclear. However, in the context of AD, the blood-brain barrier is often leaky allowing peripherally trained myeloid cells to infiltrate the CNS and potentially replace or interact with resident microglia. Third, while adjuvanted vaccines - and the AS01 adjuvant in particular - may contribute to protective effects, these effects are unlikely to be driven solely by trained immunity and may also result from the reduced pathogen-induced inflammation, mediated by adaptive immune responses (50, 51). In this context, preclinical data demonstrating improved AD-related pathology following repeated MPL injections in a mouse model (74) are consistent with a potential role for the AS01 adjuvant (50). Fourth, the extent to which trained immunity effects observed in healthy young adults translate to older, likely immunosenescent populations remains to be determined. Finally, dementia is a heterogeneous condition, and the proposed mechanisms are likely to differ across dementia subtypes; although the term ‘dementia’ is used here to encompass multiple forms, greater specificity regarding dementia subtypes will be needed to advance the field.

In the light of these limitations, it will be essential to generate additional immunological data to support or refute the hypothesis that non-specific effects of vaccination play a key role in reducing dementia risk. Such data will be critical for translating these concepts into novel therapeutic or prophylactic strategies. Notably, the recently published single-cell atlas of multiple brain regions from patients with AD, which revealed a loss of epigenomic information with disease progression, supports further investigation of epigenetic mechanisms (75). Elucidating the mechanisms underlying these promising observations may open new avenues to promote healthy ageing through vaccination and could be crucial for alleviating the global burden of dementia.

## Data Availability

The original contributions presented in the study are included in the article/supplementary material. Further inquiries can be directed to the corresponding author.
